# A multidisciplinary approach on music induced-analgesia differentiated by socio-cultural background in healthy volunteers (MOSART): A cross-over randomized controlled trial protocol

**DOI:** 10.1016/j.conctc.2024.101313

**Published:** 2024-05-22

**Authors:** Antonia S. Becker, Emy S. van der Valk Bouman, Julian Schaap, Cecile C. de Vos, Koen van Eijck, Hans Jeekel, Markus Klimek

**Affiliations:** a Department of Neuroscience, Erasmus Medical Center, Rotterdam, the Netherlands; b Centre for Pain Medicine, Erasmus Medical Centre, Rotterdam, the Netherlands; c Department of Arts and Culture Studies, Erasmus University, Rotterdam, the Netherlands; d Department of Anesthesiology, Erasmus Medical Center, Rotterdam, the Netherlands

**Keywords:** Music, Pain, Music-induced analgesia, Socio-cultural background, Cultural capital, Randomized-controlled trial

## Abstract

**Background:**

Integrating music into pain treatment demonstrates significant benefits, effectively reducing subjective pain levels and perioperative opioid requirements. Currently, the relationship between the impact of specific types of music and listeners’ socio-cultural background is still unclear. This is especially relevant given that sociological research indicates that these factors can have a notable influence on music preference and perception. Current evidence suggests that individuals who choose their own music may experience greater benefits. However, additional research is needed to comprehensively grasp whether the effect of (preferred) music on pain endurance remains consistent across different socio-cultural backgrounds.

**Methods:**

In this study, a collaborative effort between medical and sociological researchers aims to investigate music-induced analgesia differentiated by socio-cultural background in healthy volunteers. Participants (n = 84) will listen to self-, and researcher-chosen music and a podcast as a control condition in a cross-over study design. The primary outcome of this study is pain endurance measured by electric stimuli of increasing intensity. Detailed sociological validated questionnaires will be utilized. Considering the notable influence of educational level on music taste formation found in previous research and its crucial role as a source of socio-cultural differentiation, participants will be stratified based on their level of education.

**Discussion:**

This experimental study represents one of the first efforts to gain a socio-culturally differentiated understanding of the therapeutic potential of music. Consequently, this could pave the way to purposefully and inclusively implement personalized music in healthcare settings.

## Trial registration

This study was registered on www.clinicaltrials.gov with the identification number NCT06008951 in August 2023.

## 1 Background

Pain is an enormous global problem and is highly prevalent among hospitalized patients [1,2]. Pain causes significant distress to patients and is related to an increased risk of (postoperative) complications [3]. Despite increased awareness and advances in treatment options for both acute and chronic pain, effective pain management with minimal adverse events remains a persistent problem. In recent years, many studies have been published about the value of music as an adjuvant treatment for pain, also called music induced analgesia (MIA) [4].

Recorded music with the goal of ‘music as medicine’ has demonstrated benefits in different healthcare settings [[5], [6], [7]]. Examples include patients undergoing surgery, cancer patients and patients at the Intensive Care Unit (ICU) [[8], [9], [10], [11], [12]]. Additionally, evidence shows that listening to perioperative music lowers the requirement for analgesics such as opioids [13,14]. Currently, there are several theories on how music precisely affects the human body. These theories consider factors such as the influence on emotional brain centers, the autonomic nervous system, distraction, and the release of certain hormones such as dopamine and endogenous opioids [4,15]. Despite extensive research in recent years, the exact mechanism of MIA is still unclear.

Overall, there is a lack of knowledge regarding which type of music is more effective in MIA in the current literature. The description of recorded music utilized in clinical trials often lacks precision and transparency [16]. Research suggests that the patient's preferred music might be more effective in pain relief than music chosen by someone else, e.g., the researcher [[17], [18], [19]]. However, in clinical practice, allowing patients to choose their preferred music is often not possible, particularly in emergency situations or at the ICU where patients may be sedated. In those situations, health care professionals may make such decisions, reflecting their own tastes and/or in line with widespread beliefs about supposed universally effective types of music. Therefore, to purposefully use music for pain relief, it is essential to investigate the effect of different types of music, such as self-chosen and researcher-chosen music.

Currently, there is no consensus about which music works best for which patient. Previous research investigated the psychophysiological effect of self-chosen music, highlighting the importance of individual preferences [20]. While research on pain (management) is increasingly sensitive for socio-cultural differences [21,22], research on MIA notably lacks consideration for socio-cultural background characteristics such as level of education, age or ethnicity. Sociological studies have established that these characteristics – cultural capital as a trait of level of education most specifically – act as key factors contributing to musical taste differentiation [[23], [24], [25], [26], [27]] and music perception [28,29]. Moreover, these factors affect musical taste patterns, which in turn are closely aligned with listening experience and social identity, in which musical taste plays a significant role as well [30,31]. For example, people who have enjoyed higher education levels are typically much more inclined to like ‘highbrow’ music such as classical or jazz [24,26], which also prescribe different behaviors – e.g. sitting, contemplation, not singing along – during listening and concerts than less ‘legitimate’ music preferences such as rap, schlager or heavy metal [32]. As a consequence, these socio-cultural background characteristics can potentially affect MIA [26,27], which is why this should be considered when investigating the therapeutic effects of music on pain.

This study aims to investigate whether the effect of (preferred) music on pain endurance remains consistent across different socio-cultural backgrounds. With this study, we will combine knowledge from different disciplines by integrating sociological methods into medical research, which could provide new perspectives on music as medicine. The MOSART study adopts a cross-over design, wherein every participant will undergo three distinct interventions in a randomized sequence: 1) self-chosen music, 2) researcher-chosen music, and 3) a neutral spoken-word podcast as a control condition. Following each intervention, participants will be subjected to increasing electric stimuli to assess changes in pain endurance.

This study is a follow-up to a recent experimental study by our research group, in which recorded (preferred) music showed a beneficial effect over a control group with silence [33]. Building on the findings from this previous study, the current research will explore two distinct music interventions and introduce a podcast as a non-music control condition. This approach aims to specifically discern potential differences between (self- or researcher-chosen) music and any form of distraction provided by a cultural product (podcast). In this way, we aim to gain more insight into the working mechanisms of MIA specifically as opposed to other cultural interventions, and pave the way to a more personalized approach in MIA.

## 2 Methods

### 2.1 Study design

**Fig. 1:**
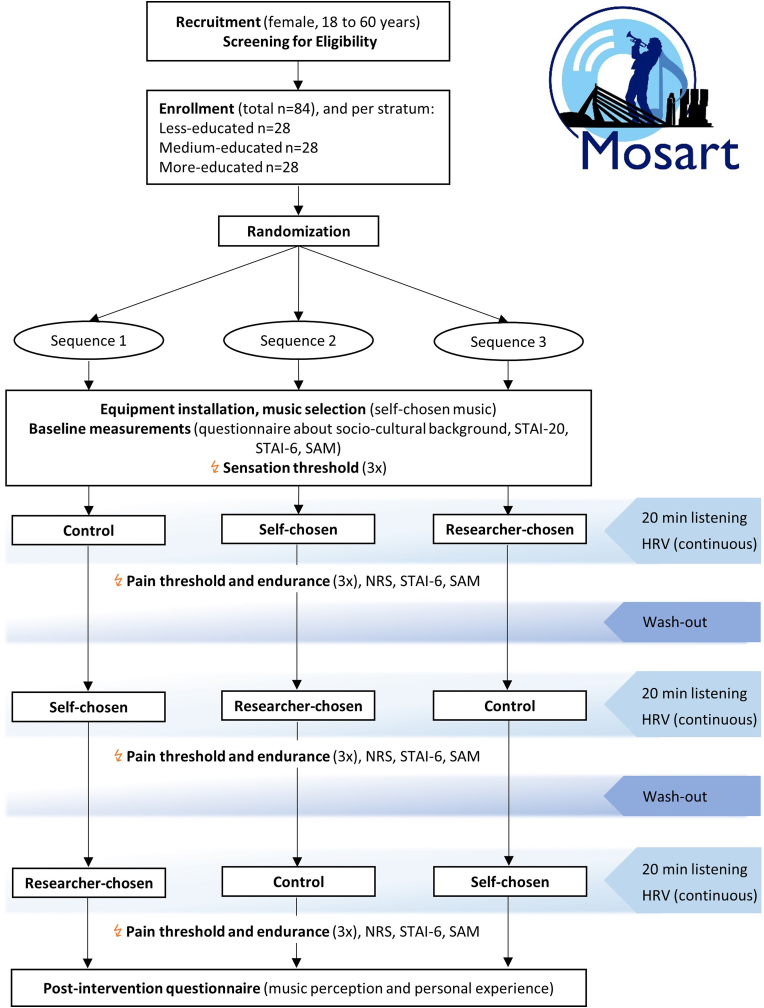
Flow diagram of the MOSART study design Note: HRV=Heart Rate Variability, NRS=Numeric Rating Scale, STAI= State-Trait Anxiety Inventory, SAM=Self-Assessment Manikin.

This randomized controlled trial investigates the effect of self-chosen music and researcher-chosen music while considering socio-cultural background. Listening to a neutral, non-musical informative podcast will serve as a non-music control condition. The study will have a Latin-square crossover design to account for individual differences in pain perception and the influence of music. Hence, each participant will receive all three interventions (self-chosen music, researcher-chosen music, and control) in a randomized order. Each intervention has a duration of 20 min, and there will be a 20-min washout period between interventions. In this wash-out period, no music or stressful activities will be allowed. At the end of each intervention, pain endurance by increasing electric stimuli will be tested as the primary outcome of this study. Moreover, physiological assessments, psychological questionnaires – evaluating emotions and anxiety – and a comprehensive survey regarding socio-cultural background will be conducted. Measurements will take place at the outpatient clinic of the Center of Pain Medicine at Erasmus Medical Center, Rotterdam. See Fig. 1 for a comprehensive overview of the study design.

### 2.2 Sample size calculation

As there is currently no published research examining the interaction of music with socio-cultural background in relation to pain sensation, we conducted a power analysis based on the same meta-analysis that was used in the previous trial (CRESCENDo-trial) [5,33]. We estimated a slightly lower effect size (Cohen's d 0,4), considering the utilization of a podcast as a non-music control condition in this study. The intraclass correlation coefficient (ICC) was estimated based on a study that assessed pain intensity and unpleasantness ratings before and after listening to music [17]. To obtain a power of 90 % with a two-sided significance level of 5 % and under consideration of possible dropouts, we calculated that a sample size of 84 was needed. Thus, each group, stratified by level of education, will consist of n = 28.

### 2.3 Procedures

#### 2.3.1 Stratification and randomization

Current sociological literature provides evidence that humans are differently socialized regarding music taste and music perception [[23], [24], [25], [26], [27],[34], [35], [36]]. Stratified recruitment and randomization (Castor EDC, Ciwit B·V., Amsterdam) based on level of education will be performed to ensure maximum variation in educational levels in our sample. Since level of education is a key indicator of cultural capital [37], which in turn plays a significant role in shaping individual musical preferences, stratification will be based on participants’ level of education [34]. Thus, the study population will consist of 1/3 less-educated, 1/3 medium-educated, and 1/3 more-educated female subjects. This categorical strategy was chosen because other groupings, such as years of education, are less suitable in the Dutch education system, where students are separated into different educational tracks at the age of 12. A comparison between these three categories will allow us to compare participants belonging to clearly defined educational groupings and thus assess the impact of schooling level as a proxy for cultural capital. An overview of the stratification strategy by level of education can be found in Appendix A.

#### 2.3.2 Exclusion and inclusion criteria

Heterogeneity in pain tolerance is partly linked to gender and age [38,39]. Therefore, including only female subjects between the ages of 18 and 60 years will create a more homogenous study population. The upper age limit of 60 years also aligns with the observation that responsiveness in autonomic activity significantly decreases in the elderly, which could influence Heart Rate Variability (HRV) measurements [40,41]. Furthermore, the usage of analgesic medication within the last 24 h and/or chronic and acute pain conditions were exclusion criteria in this study. A full overview of the in- and exclusion criteria can be found in Table 1.

**Table 1:** Overview of the eligibility criteria.

Inclusion criteria	Exclusion criteria
• Between 18 and 60 years of age	• Significant hearing impairment
• Female	• Current complaints of tinnitus
• Sufficient knowledge of the Dutch language	• Current treatment by a medical specialist or general practitioner
• Provision of written informed consent	• Current use of analgesic medication
	• Presence of acute or chronic pain
	• History of cardiac disease of arrhythmias
	• Electric implants (e.g. pacemakers)
	• Diagnosed psychiatric or neurological impairments
	• (Suspected) pregnancy

#### 2.3.3 Music and control interventions

Most studies on music interventions show high heterogeneity in music selection. Based on earlier research, it is challenging to determine the optimal combination of music and setting that works best for each individual [16]. Some evidence suggests that having more freedom to choose music and listening to preferred music is superior [[17], [18], [19],^42^]. Moreover, several (clinical) studies exclusively use classical and/or ‘relaxing’ ambient music, following the cultural belief that this music might be superior in reducing pain [[43], [44], [45]]. However, no substantial evidence supporting this belief has been found so far. Therefore, subjects will receive two different types of music intervention (self-chosen and researcher-chosen) and a neutral, informative podcast as a non-music control condition. Each intervention will last 20 min before pain endurance is tested. Standardized instructions will be utilized to introduce the three interventions and explain the study procedure to the participants. Due to the nature of the study and the music intervention, it is not possible to blind participants and investigators.

The self-chosen music will be selected by the participants in advance. The researcher-chosen playlist will be created by the Music as Medicine research group from Erasmus Medical Center. The playlist will be created with the goal of reducing pain perception based on previous literature [5,18,26,46]. An overview of the researcher-chosen music can be found in Appendix B. The selection of the podcast is guided by two factors: 1) ensuring the content is entirely non-musical (i.e., comprising only voice or conversation), and 2) ensuring the content is neutral, devoid of political, ideological, or cultural subjects. Therefore, participants will be listening to an informative podcast about flora and fauna which makes no mention of climate change or other obvious topics that may elicit strong emotional reactions and/or political polarization.

### 2.4 Measurements

#### 2.4.1 Pain endurance

The primary outcome of this study is pain endurance, quantified in amperage, through increasing electric stimuli administered at the end of each intervention (self-chosen music, researcher-chosen music, and control). The electric stimuli will be administrated by the STMISOLA (Biopac Systems Inc. Goleta, CA, USA). Two electrodes will be attached to the index finger of the non-dominant hand. Administration of the electrical pulses with a frequency of 100 Hz will start when the participants press a button with the other, dominant hand. When this button is pressed, electric pulses with a current increase rate of 0.5 mA/s will be administered up to a maximum of 30 mA for safety reasons.

When the experiment starts and the participant is connected to the device, the sensation threshold will be tested. At the end of each intervention, while the participants are still listening to music or the podcast, the pain endurance will be tested. Standardized instructions will be used, instructing participants to tolerate the electric pain stimuli as long as possible. Each measurement will be repeated three times, and the average amperages will be registered.

#### 2.4.2 Physiological assessment

Heart rate variability (HRV) will be monitored continuously throughout each listening intervention and pain stimuli procedure. HRV, the variation in time between consecutive heartbeats, can be used as a marker for autonomic function [47,48]. An increase in HRV can be seen due to activation of the parasympathetic nervous system, causing relaxation and recovery [49]. This effect can also be seen when listening to music [50]. HRV will be measured using an Acentas Chest Strap (BM innovations GmbH), providing an objective measure of the physiological response to the interventions [51].

#### 2.4.3 Psychological questionnaires

In addition to the objective pain endurance, as measured in amperage, participants will be asked to rate the pain intensity and pain unpleasantness using an 11-point Numeric rating scale (NRS), where 0 implies no pain and where 10 implies the worst pain imaginable [52]. Since pain is a highly subjective experience, measuring (changes in) emotions is essential in pain assessment [53]. Anxiety will be measured with the State-Trait Anxiety Inventory (STAI-6) at baseline and after each intervention [54,55]. This questionnaire consists of six items and is a validated and frequently used method (Cronbach's α 0.83) [56]. Furthermore, the participants' emotions will be assessed with the Self-Assessment Manikin (SAM) at baseline and after each intervention. The SAM is a validated non-verbal pictorial assessment technique that directly assesses the emotional valence (Cronbach's α 0.73) and arousal (Cronbach's α 0.98) associated with a person's affective response to a wide variety of stimuli [57,58]. Moreover, the personal experience of listening to music and the podcast will be evaluated at the end of the experiment with five open-ended questions. This evaluation aims to account for confounders and to elaborate upon any interaction(s) between personal experience, music perception and pain endurance.

#### 2.4.4 Socio-cultural background

This study will assess socio-cultural background of participants with validated sociological questionnaires, focusing on participants' cultural capital (Cronbach's α 0.84) and parental cultural capital (Cronbach's α 0.73) [59]. Before the experiment starts, participants will fill out a comprehensive questionnaire, which assesses level of education, occupation, income, ethnicity and participants' and participants' parental cultural capital. Since parental socioeconomic status is a strong determinant of early socialization, which affects both embodied cultural capital (taste) and institutionalized cultural capital (level of education) [23,60], parents' income, level of education and cultural capital also serve as strong indicators of participants' socio-cultural background and will therefore be assessed in this study [61,62]. Hence, socio-cultural background will be assessed using these variables: level of education (participant and parents), current occupation, income (participant and parents), and cultural capital (participant and parents). Current occupation and household income will be grouped according to the Goldthorpe class schema [63].

Additional baseline characteristics such as age, medical history and everyday music listening behavior will be evaluated. Music listening behavior will be assessed as part of the participant's cultural capital. The participant is asked about weekly hours dedicated to passive and active music listening, importance attributed to music and whether they play any instruments and/or are professional musicians. An overview of the questionnaire about background characteristics and socio-cultural background is provided in Table 2.

**Table 2:** Overview of the questionnaire about socio-cultural background.

Questionnaire groups	Questionnaire items
General background	Age Handedness (right- or left-handed) Length and weight Earlier participation in comparable experiment(s)
Medical questions	Medical history Usage of medication Usage of alcohol Usage of drugs
Economic status	Current occupation (*participant*) Monthly income (*participant, parents*)
Cultural capital	Level of education of (*participant, parents*) Frequency of the following activities (*participant, parents*) Classical concert/opera/ballet Museum of arts Theater Cinema Dining out Pop-/rock concert Visiting sports events Night out with friends/family Hiking/biking Importance of art and culture (*participant, parents*) Importance of music Active music listening per day Passive music listening per day
Ethnicity	Country of birth (*participant, mother, father*)
	

#### 2.4.5 Music characteristics

Literature indicates that different elements of music such as tempo and genre may have varying effects on MIA [46,64,65]. Our research group is interested in which music people choose to alleviate pain and how music selection is affected by socio-cultural background. Therefore, the self-chosen and researcher-chosen music will be assessed and compared with the help of Spotify® Application Programming Interface (API).

### 2.5 Data management and statistical analysis

Data will be managed using Castor EDC (Ciwit B·V., Amsterdam), a certified data capture tool that includes skips, validation checks and an audit trail. This study will look at the influence of (preferred) music on MIA across different socio-cultural backgrounds (through an explorative approach. For normally distributed data, we plan to utilize a linear mixed model to analyze both the primary outcome (pain endurance) and secondary outcomes (pain intensity and unpleasantness, HRV, SAM, STAI-6). In the fixed-effects part of the model the following effects will be included: intervention, position within randomization sequence, age, TRAIT anxiety, level of education, economic capital (income), participants' cultural capital, parents’ cultural capital and ethnicity. Interaction terms such as intervention and position within randomization sequence will eventually be included using F and likelihood ratio test. In the random-effects structure we will incorporate random intercepts and a random effect of the time point of sequence. Because of the continuous measurement of HRV during each intervention the time point (within each intervention) will be included as fixed and random effect. This way, we will consider the period effect and potential carry-over effects of the cross-over study design. In the event of not normally distributed data a Friedman test will be performed instead. Moreover, subgroup analyses excluding participants with maximal pain endurance during all three interventions and non-adherence to the protocol will be performed. Data analysis will be performed using R version 4.0 or higher.

### 2.6 Ethical approval and trial status

The study protocol received ethical approval from the Medical Ethical Review Committee of the Erasmus Medical Center in Rotterdam (registration number MEC-2023-0253) on July 31, 2023, before the start of the study. This study will be conducted according to the principles of the Declaration of Helsinki (64th WMA General Assembly, Fortaleza, Brazil, October 2013) and in accordance with the Medical Research Involving Human Subjects Act. The trial is registered with the National Institute of Health (www.clinicaltrials.gov, ID: NCT06008951). All participants will be asked for written informed consent and will receive financial compensation for their time investment.

This study protocol (version 2.1, October 24th, 2023) follow the SPIRIT (Standard Protocol Items: Recommendations for Interventional Trials) guideline [66] and the CONSORT (Consolidated Standards of Reporting Trials) guidelines for non-pharmacological treatments [67].

## 3 Discussion

### 3.1 Bridging sociological and medical research

This study will be the first experimental trial that integrates sociological theories and methods into medical research on MIA, which could provide new perspectives on music as medicine. As a result of this collaboration, we aim to assess the effects of a range of relevant socio-cultural background indicators on MIA. Sociological research indicates, among other findings, that music preference correlates with socio-cultural background characteristics, including gender, ethnicity, cultural capital, and economic status. Furthermore, the chosen music can influence individuals' behavior, actions, and emotions [26,27]. As pain is defined as an “unpleasant sensory and emotional experience,” it is expected that different music preferences will influence emotions and thereby also the experience of pain [53]. Moreover, it seems probable that music influences pain through neurobiological processes, such as the activation of the parasympathetic nervous system and the release of endogenous neurotransmitters. These effects may also depend on music perception and socio-cultural background characteristics [4,50].

The MOSART study was designed as a collaboration between the medical research group ‘Music as Medicine’ of the Erasmus Medical Center Rotterdam and the ‘Rotterdam Popular Music Studies’ group of the Erasmus University Rotterdam. This sociological research group focuses on the reception of music in relation to social background characteristics. The medical research group specializes in investigating the physiological and health-related effects of music, including MIA and its influence on the autonomic nervous system, as measured with HRV. We believe in providing a unique perspective that enhances our comprehension of how socio-cultural background contributes to MIA.

### 3.2 General aspects and considerations about the study

#### 3.2.1 Relation between pain endurance, music and socio-cultural background

Previous research indicates a crucial role of socio-cultural background (level of education and cultural capital) in music taste formation and music perception [[23], [24], [25], [26], [27], [28]]. Thus, while it is clear that people with different backgrounds have different musical tastes, it is not clear to what extent the impact of music on pain endurance depends on these features. We hypothesize two contrasting possibilities.

First, we hypothesize that the impact of music largely depends on the degree to which it aligns with personal taste, as previous research indicates [18,19]. People will then respond most to the type of music they actually appreciate and benefit less from music that leaves them indifferent or even annoys them. In this scenario, music that fits participants’ taste will enhance pain endurance more than non-preferred music. If personal taste is indeed important, irrespective of the exact taste, we expect its effect to be similar across groups with different socio-cultural backgrounds. Our first hypothesis is therefore that the positive impact of self-chosen, preferred music on MIA is consistent for all participants.

Second and in contrast, if researcher-chosen music has a universal beneficial impact, then people's (socially differentiated) taste should not affect the degree to which music enhances pain endurance. In other words: the impact of taste should be absent, irrespective of one's socio-cultural background, demonstrating that some types of music are simply more effective in providing MIA than others. In this scenario, our second hypothesis is then that the positive impact of researcher-chosen music on MIA is consistent for all participants, independent of their socio-cultural backgrounds.

#### 3.2.2 Conceptual basis of time periods used within study design

The length of the (music) listening periods were based on previous clinical and experimental studies [5,33,68]. Although clinical trials show especially high discrepancies in the length of music listening, ranging from 5 to 120 min, the most frequent listening durations last between 15 and 30 min [16,69]. Therefore, we chose to incorporate a 20-min period for all three interventions (self-chosen music, researcher-chosen music and control condition).

The choice of a 20-min wash-out period was grounded in the pharmacokinetics of potential key neurochemicals implicated in MIA. Given the incomplete understanding of the mechanisms underlying MIA, we aimed for the wash-out period to be strategically positioned relative to the half-lives of potential key neurochemicals. Studies have highlighted the involvement of neurotransmitters such as dopamine, beta-endorphin and oxytocin in modulating pain perception in MIA [[70], [71], [72]]. The half-life times of these specific neurotransmitters have a broad range from 1 min to 1.5 h, but predominantly last from a couple of minutes to 20 min [[73], [74], [75]]. By allowing a 20-min wash-out period between the interventions in this study, we aim to minimize carryover effects. This rationale aligns with the principles of crossover study design, facilitating the accurate assessment of treatment effects while controlling for potential confounders and randomization sequence. Additionally, the choice of a 20-min duration aims to balance the need for an adequate wash-out period with considerations for volunteer comfort and study feasibility.

### 3.3 Challenges of the study

In the MOSART study, we aim to ensure a diverse study population with varied socio-cultural backgrounds. This emphasis is crucial because inclusive medical practice demands that we focus on more diverse social backgrounds, distinct from the typically prevalent highly educated Western study cohorts that have been overrepresented in prior experimental research on MIA [13]. Therefore, we are opting to employ stratification based on education level, recognizing it as one of the most important indicators for socio-cultural background. According to earlier sociological research experience, it can be challenging to include volunteers with lesser education levels in experimental studies in the Netherlands. Therefore, we are planning to expand communication strategies to reach more potential participants, especially from less- and medium-educated strata.

Another challenge can be found in the multidisciplinary aspect of this study. This study is based on collaboration among the interdisciplinary research group with researchers from the departments of anesthesiology, surgery and neuroscience, and sociology. A challenge in this collaboration is that the research methods can differ, for instance in study design and sample size. For example, to combine a manageable sample size that aligns with sociological aspects (higher sample sizes) with relatively time-demanding inclusions common in medical research (lower sample sizes), we are choosing an exploratory approach toward analyzing sociological variables. Moreover, by only including female participants, we aim to establish a more homogenous study population so that drawing valid conclusions about the effects of the socio-cultural background indicators remains feasible. This is particularly important as gender differences – especially the gender of researchers and participants – are strongly linked to pain perception [38,[76], [77], [78]]. Therefore, only female participants and researchers will be involved in this study. Finally, given that pain and music preference are multidimensional concepts, we contend that a comprehensive understanding of our research question can only be achieved by combining various disciplinary insights that are integral to the design of this research protocol.

## Trial status

First enrollment took place on 30 Augustus 2023. Currently, on March 1st^,^ 2024, a total of 50 participants were enrolled.

## Funding

The MOSART study is financially supported by the Erasmus MC Foundation.

## Data availability

No data was used to write this article.

## CRediT authorship contribution statement

**Antonia S. Becker:** Writing – review & editing, Writing – original draft, Visualization, Methodology, Conceptualization. **Emy S. van der Valk Bouman:** Writing – review & editing, Writing – original draft, Visualization, Methodology, Conceptualization. **Julian Schaap:** Writing – review & editing, Writing – original draft, Methodology, Conceptualization. **Cecile C. de Vos:** Writing – review & editing, Methodology. **Koen van Eijck:** Writing – review & editing, Methodology, Conceptualization. **Hans Jeekel:** Writing – review & editing, Supervision. **Markus Klimek:** Writing – review & editing, Supervision, Methodology, Conceptualization.

## Declaration of competing interest

The authors declare that they have no known competing financial interests or personal relationships that could have appeared to influence the work reported in this paper.
