# Supplementary material for: A multidisciplinary approach on music induced-analgesia differentiated by socio-cultural background in healthy volunteers (MOSART): A cross-over randomized controlled trial protocol
Source: Contemp Clin Trials Commun. 2024 May 22;39:101313. doi: 10.1016/j.conctc.2024.101313 (PMC11179059; doi:10.1016/j.conctc.2024.101313)
Supplement: Multimedia component 1 [file mmc1.docx]

**APPENDIX A – Stratification strategy by level of education**

| **Level of education** | **Education types** | **Dutch education equivalent** |
| --- | --- | --- |
| Less-educated | Primary education, prevocational secondary education | Basisonderwijs, VMBO |
| Moderate-educated | Senior secondary/ general vocational education, pre-university education | MBO, HAVO, VWO |
| More-educated | Higher education university, higher vocational education | WO, HBO |

Note: The Dutch education system was used to categorize all the participants’ level of education (1, 2). The letters in brackets are Dutch abbreviations of different education levels. MAVO = Middelbaar Algemeen Voortgezet Onderwijs. VMBO = Voorbereidend Middelbaar Beroepsonderwijs. HAVO = Hoger Algemeen Vormend Onderwijs. VWO = Voorbereidend Wetenschappelijk Onderwijs. HBO = Hoger Beroepsonderwijs. WO = Wetenschappelijk Onderwijs

**APPENDIX B – The ‘researcher-chosen music’**

| **Song** | **Artist** | **Length** | **BPM** | **Popularity** | **Key** |  |
| --- | --- | --- | --- | --- | --- | --- |
| Sonata No. 14 "Moonlight" in C-Sharp Minor", Op. 27 No. 2: I. Adagio sostenuto | Ludwig van Beethoven, Paul Lewis | 5:15 | 86 | 71 | C# minor |  |
| Cello Suite No. 1 in G Major, BWV 1007: I. Prélude | Johann Sebastian Bach, Yo-Yo Ma | 2:32 | 75 | 68 | G major |  |
| Clarinet Concerto in A major, K.622: II. Adagio | Wolfgang Amadeus Mozart – Martin Fröst, Deutsche Kammerphilharmonie Bremen | 6:40 | 67 | 49 | D major |  |
| Concerto in D Minor, BWV 974: II. Adagio | Johann Sebastian Bach, Víkingur Ólafsson | 4:10 | 64 | 47 | D minor |  |
| Nocturne No. 2 in E-Flat Major, Op. 9 No. 2 | Frédéric Chopin, Daniel Barenboim | 4:34 | 79 | 68 | Eb major |  |
| Minuet in A major, D. 334 | Franz Schubert, Arcadi Volodos | 3:13 | 92 | 64 | A major |  |

The researcher-chosen music was selected based by our research group and partly based on characteristics predominantly described in the literature. Therefore, only songs with a BPM of less than 100 and higher popularity, accounting for the possible preference for favorite music, were included. Moreover, since sociological research has indicated that classical music is generally well-received and to ensure uniformity, classical popular music was chosen (3). Length, BPM, Popularity and Mode were determined with the Spotify® Application Programming Interface.

Note: BPM= beats per minute

**References in Appendix**

1. Centraal Bureau voor de Statistiek. Standaard Onderwijsindeling 2021 Editie 2022/’23. 2021 [updated November 16 2023. Available from: <https://www.cbs.nl/-/media/cbs/onze-diensten/methoden/classificaties/documents/2023/pubsoi2021_ed2223.pdf>.

2. Limacher U. Dutch education explained by Genoveva Geppaart. Ute’s International Lounge & Academy. [updated 16 October 2023. Available from: <https://utesinternationallounge.com/dutch-education-explained-by-genoveva-geppaart/>.

3. Bryson B. "Anything But Heavy Metal": Symbolic Exclusion and Musical Dislikes. American Sociological Review. 1996;61(5):884-99.
